# Chiral Alkyl Halides: Underexplored Motifs in Medicine

**DOI:** 10.3390/md14110206

**Published:** 2016-11-04

**Authors:** Bálint Gál, Cyril Bucher, Noah Z. Burns

**Affiliations:** Department of Chemistry, Stanford University, 333 Campus Drive, Stanford, CA 94305, USA; bgal@stanford.edu (B.G.); cbucher@stanford.edu (C.B.)

**Keywords:** alkyl halide, stereochemistry, lipophilicity, anesthetic, clindamycin, corticosteroid, pimecrolimus, sucralose, halomon, lapachone, astins, forazoline

## Abstract

While alkyl halides are valuable intermediates in synthetic organic chemistry, their use as bioactive motifs in drug discovery and medicinal chemistry is rare in comparison. This is likely attributable to the common misconception that these compounds are merely non-specific alkylators in biological systems. A number of chlorinated compounds in the pharmaceutical and food industries, as well as a growing number of halogenated marine natural products showing unique bioactivity, illustrate the role that chiral alkyl halides can play in drug discovery. Through a series of case studies, we demonstrate in this review that these motifs can indeed be stable under physiological conditions, and that halogenation can enhance bioactivity through both steric and electronic effects. Our hope is that, by placing such compounds in the minds of the chemical community, they may gain more traction in drug discovery and inspire more synthetic chemists to develop methods for selective halogenation.

## 1. Introduction

Alkyl halides are among the most versatile compounds in the chemical industry. Small haloalkanes are some of the most commonly used solvents in chemical laboratories; chlorofluorocarbons have seen widespread use as refrigerants and propellants; and compounds containing both Br and F are often used in fire retardants. Within synthetic organic chemistry, they are commonly used in alkylation reactions, radical cascades, and alkyl cross-coupling chemistry [1,2,3].

**Figure 1:**
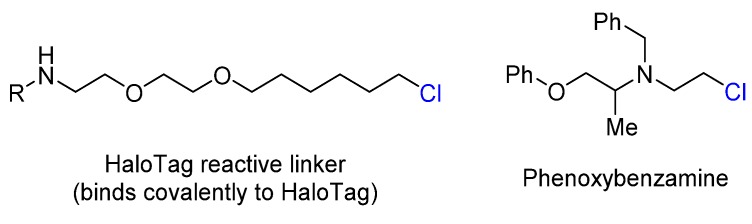
The structures of HaloTag and Phenoxybenzamine.

The reactive nature of primary alkyl chlorides is sometimes exploited in medicinal chemistry and chemical biology. HaloTag is a modified haloalkane dehalogenase that is used to covalently bind to a synthetic ligand of choice and fuse to a protein of interest (Figure 1) [4]. This construct allows for a variety of experiments including protein purification, protein stability studies and protein translocation assays. This technology relies on the use of an alkyl chloride linker that rapidly (typically within minutes) forms a covalent bond with the protein tag under physiological conditions. Phenoxybenzamine, an irreversible α-blocker, forms covalent bonds with adrenergic receptors through the attack of a cysteine residue at the alkyl chloride moiety in transmembrane helix 3 (Figure 1) [5].

While primary alkyl halides are often reactive for displacement by nucleophiles, secondary and tertiary chlorides and bromides are significantly less reactive. As a testament to their stability, six drugs on the World Health Organization’s essential medicines list contain such motifs [6].

A key consideration in drug development is the pharmacokinetic profile and efficacy of a drug candidate [7]. Halogenation of sp^2^ carbons is commonly used to increase lipophilicity, which can lead to improved membrane permeability and oral absorption [8]. Furthermore, halogenation can also enhance the blood–brain barrier permeability, which is crucial for drugs targeting the central nervous system [9]. These properties are most often exploited by the introduction of aryl chlorides into drug candidates [10]. Indeed, approximately a third of the compounds in clinical trials contain these motifs. Intriguingly, alkyl chlorides are only used in a handful of drugs, some of which are described below.

This review aims to detail some compounds of medicinal significance that contain a halogen-bearing stereogenic center. We hope to illustrate the importance of these motifs and the roles they play to enhance the biological and pharmacological properties of drugs. Fluorine, due to its small size and high electronegativity, imparts properties on molecules that are unique to this element. As these effects have been reviewed previously [11], herein we will focus on alkyl chlorides and bromides.

## 2. Alkyl Halides in Medicine

### 2.1. Anesthetics

**Figure 2:**
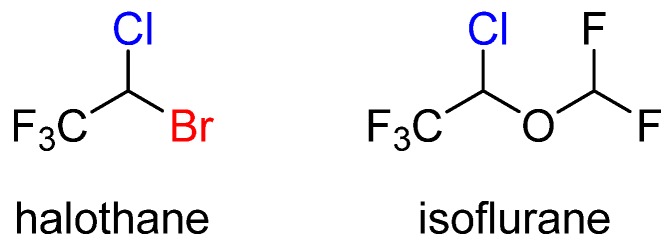
The structures of halothane and isoflurane.

General anesthetics are agents that can cause reversible loss of consciousness. They have been used in medicine since the mid-1800s [12], however their mechanism of action remains a topic of debate. For decades, the general view was that anesthetics act by nonspecific perturbation of lipid membranes. This hypothesis has more recently been disproven as a result of a number of findings. Most importantly, stereospecific effects have been observed for general anesthetic binding. Two chiral alkyl halide anesthetics, isoflurane and halothane, are on the WHO’s list of essential medicines (Figure 2) [6].

In 1991, N. P. Franks and W. R. Lieb found that the two optical isomers of isoflurane exhibited differential binding to ion channels in identified molluscan central nervous system neurons [13]. The (+)-isomer was roughly twofold more effective than the (−)-isomer at eliciting the anesthetic-activated potassium current *I*_K(An)_ at the human median effect dose (ED_50_) for general anesthesia. Both isomers were found to be equally effective at disrupting lipid bilayers.

It is now postulated that general anesthetics exert their action through the activation of inhibitory central nervous system (CNS) receptors, and through the inactivation of CNS excitatory receptors [12]. Recently, a small number of molecular targets have emerged. These include gamma-aminobutyric acid type A receptors (GABA_A_), two pore domain potassium (2PK) channels, and *N*-methyl-d-aspartate (NMDA) receptors. How anesthetics interact with these targets on a molecular level is also becoming clearer.

**Figure 3:**
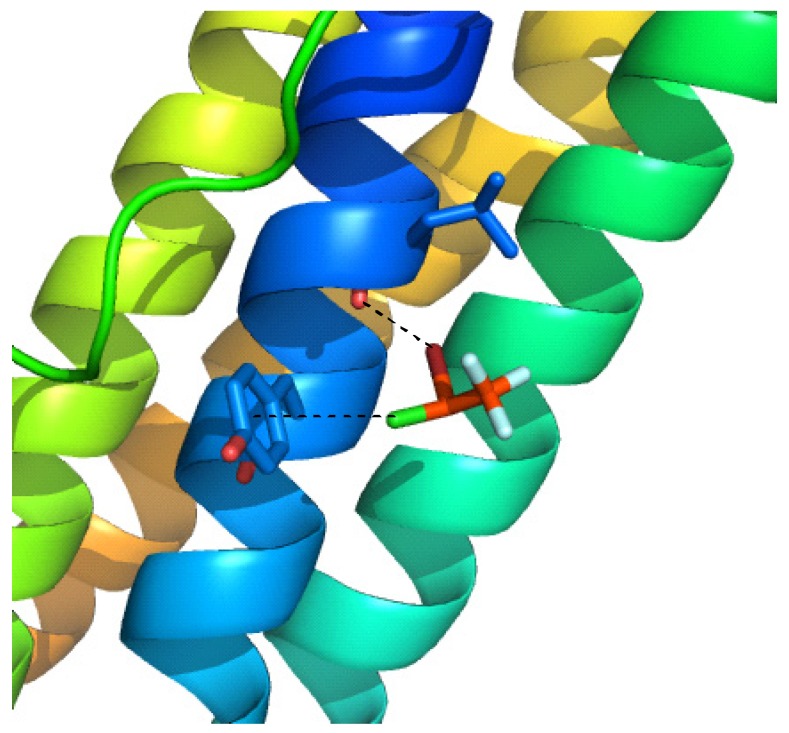
Complex of halothane with apoferritin. The key binding interactions include halogen bonds between Br and the carbonyl oxygen of Leu24, and Cl-π interaction between Cl and Tyr28.

One protein that halogenated anesthetics have been shown to interact with is ferritin, a large 4-helix bundle protein. In 2005, Liu and coworkers solved the structure of both halothane/ferritin and isoflurane/ferritin complexes (Figure 3) [14]. Halothane binds in a hydrophobic cavity between two ferritin monomers. While racemic compound was used for this study, the electron density map revealed that the (+)-isomer was twice as abundant as the (−)-isomer. Due to the hydrophobic environment, the strongest contributors to binding are halogen bonding interactions [15] between the Br of halothane and ferritin’s carbonyl oxygen at Leu24, as well as a halogen-π contact between halothane’s Cl atom and the aromatic ring in Tyr28 [16], Figure 3. These results very clearly illustrate that the stereochemistry of alkyl halides matters for binding interactions, and that halogen atoms can provide key binding interactions.

### 2.2. Clindamycin

**Figure 4:**
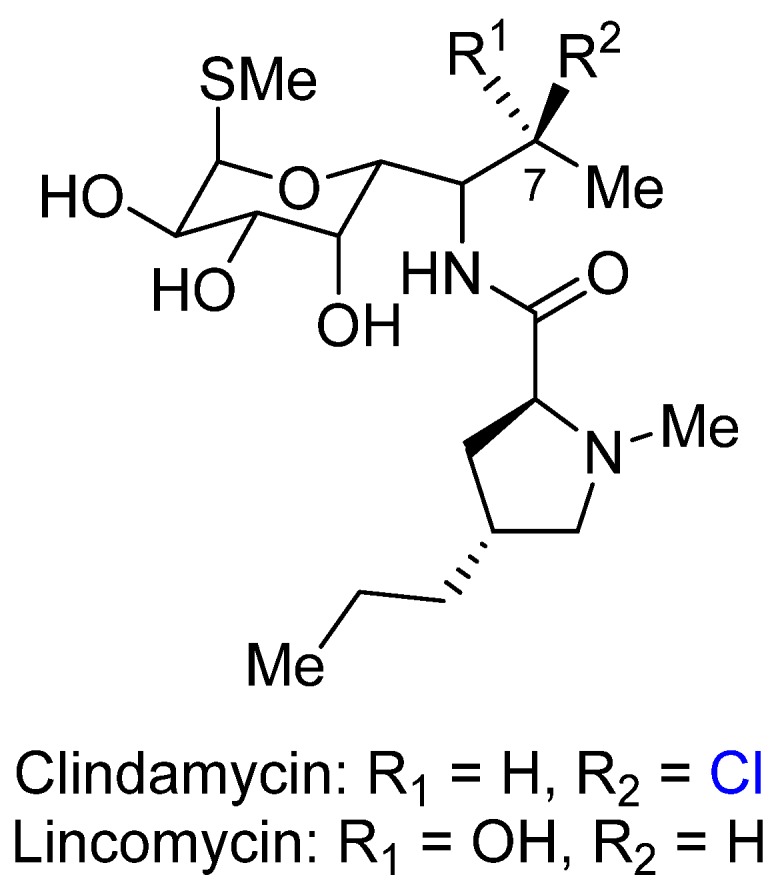
The structures of lincomycin and clindamycin.

Clindamycin is a broad spectrum antibiotic derived semisynthetically from the natural product lincomycin (Figure 4). Clindamycin is used for the treatment of a variety of bacterial infections, including bone and joint infections, strep throat, pneumonia, and endocarditis [17]. Similarly to macrolide antibiotics, lincomycin and clindamycin inhibit protein synthesis by ribosomal translocation—they bind to the 50S rRNA of the large bacterial ribosome subunit [18].

In 1985, Francois Le Goffic investigated structure-activity relationships of lincomycin analogs [19]. They found that clindamycin is highly active compared to lincomycin against a number of test organisms, while having lower toxicity levels. A number of derivatives at the 7 position have been synthesized and tested (see Table 1). The results indicate that in vivo potency is increased when the hydrophilic hydroxyl groups are substituted with chloride or bromide. When the stereochemistry of C7 is swapped, the efficacy is increased. This clearly shows the importance of the stereochemistry of chiral alkyl halides for biological activity. Interestingly, substituting chlorides with bromides and iodides further increased activity. These results clearly demonstrate that halogenation can enhance efficacy in vivo, and that the exact stereochemistry can play an important role for bioactivity.

**Figure 5:**
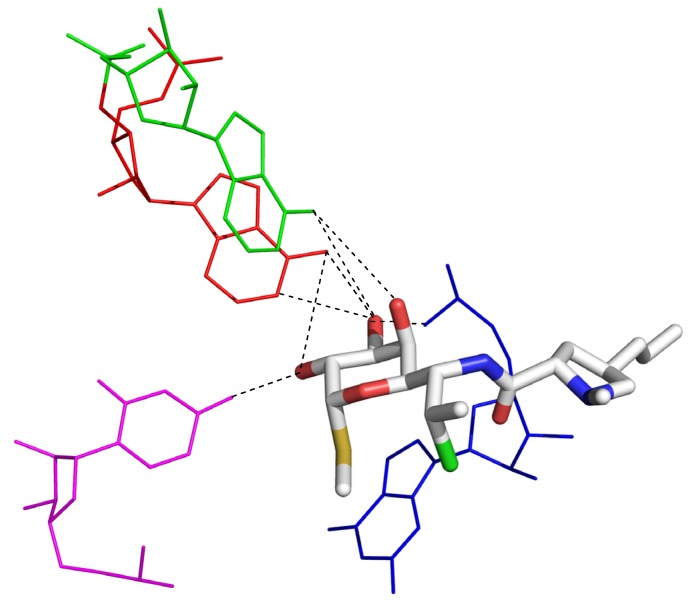
Crystal structure of clindamycin bound to the peptidyl transferase cavity of the 50S subunit. The key binding interactions are provided by the sugar hydroxyl groups.

To better understand these differences between lincomycin and clindamycin on a molecular level, further studies were conducted to elucidate the binding properties. In 1992, the interaction of clindamycin and lincomycin with *E. coli* 23S ribosomal RNA was investigated by chemical footprinting [20]. The in vitro binding affinities of the two drugs for the ribosome were found to be roughly the same (*K*_diss_ = 5 μM for lincomycin and 8 μM for clindamycin), even though the exact geometries of binding were somewhat different. The structure of a complex between clindamycin and the 50S ribosomal subunit of the eubacterium *Deinococcus radidurans* was solved in 2001 (Figure 5) [21]. The crystal structure revealed no strong interaction with the Cl atom of clindamycin. Instead, the key binding interactions arise from hydrogen bonding interactions between the hydroxyl groups of the sugar moiety and nucleosides. Both of these studies concluded that the higher activity of clindamycin was not attributable to stronger binding, but to increased lipophilicity. However, lipophilicity alone cannot explain the differential activity of C7 stereoisomers. Further research would be instructive in elucidating how the chloride stereochemistry exactly bears on bioactivity.

### 2.3. Corticosteroids

**Figure 6:**
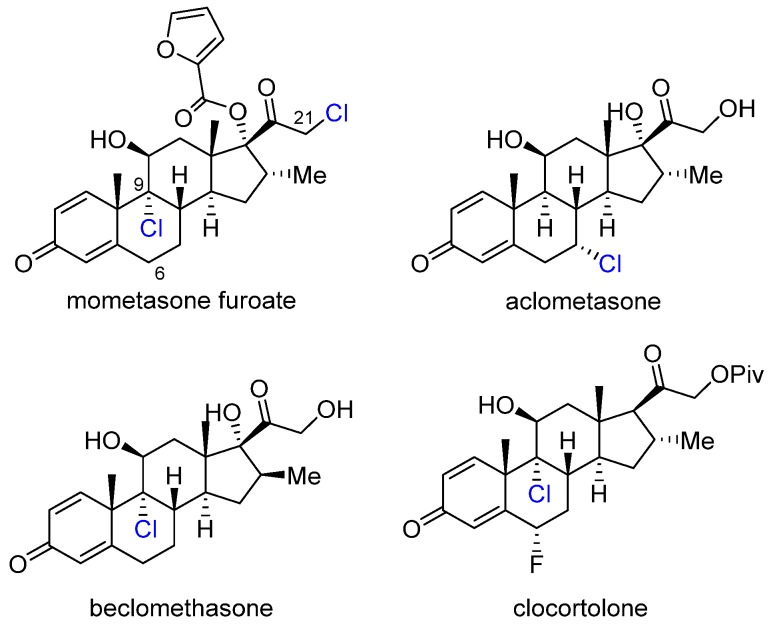
The structures of chlorinated corticosteroids used in medicine.

Corticosteroids are among the most widely used drug classes due to their ability to exert intense biological effects in almost any organ (Figure 6). They are often the drug of choice for their anti-inflammatory and immunosuppressive properties [22]. These steroids act by binding to glucocorticoid receptors (GRs), therefore the receptor-binding affinity is a major determinant of therapeutic potential [23].

In the case of mometasone furoate, it was found that there is a dipole–dipole interaction between the C21 Cl and an asparagine residue in the binding pocket AncGR2-Asn33 that contributes to the high affinity of binding [24].

To correlate other structural features with binding affinity to glucocorticoid receptors, extensive structure activity relationship studies were carried out on this class of compounds [25,26]. A key finding was that fluorination or chlorination leads to 6–7-fold increase in binding affinity. Interestingly, bromination at the same positions actually reduces binding, which implies a size limitation for effective fit in the binding pocket. The origin of these effects is unknown at this stage, further research is needed to understand how halogenation can have such a striking effect on binding affinity and in turn drug efficacy.

### 2.4. Pimecrolimus

**Figure 7:**
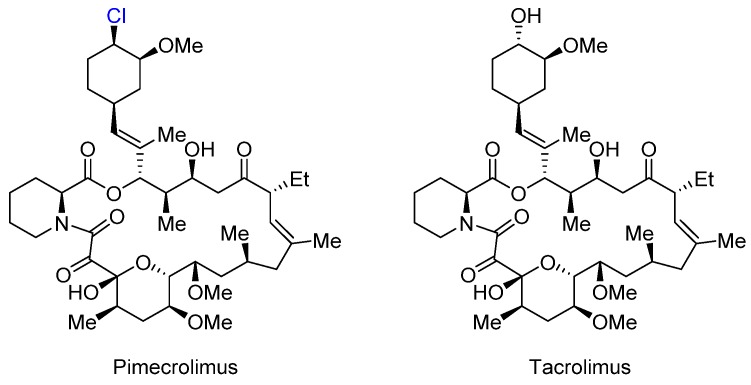
The structures of Pimecrolimus and Tacrolimus.

Pimecrolimus and tacrolimus are immunosuppressants used for the topical treatment of atopic dermatitis (eczema). They both bind to the protein macrophilin-12 and inhibit the phosphatase calcineurin, resulting in the blockage of T cell activation (Figure 7) [27].

Despite a high degree of similarity in structure and mechanism of action, pimecrolimus and tacrolimus display characteristic differences in terms of pharmacological profile [28]. As a result of OH to Cl substitution, pimecrolimus is approximately eight times more lipophilic based on the octanol/water distribution coefficient (Elog*D*_oct_ = 6.99 ± 0.05 for pimecrolimus vs. 6.09 ± 0.04 for tacrolimus). Consequently, it has a higher affinity to the skin and lower levels of permeation. This explains the observed lower systemic exposure to pimecrolimus than to tacrolimus after topical application. The unbound fraction of pimecrolimus in human plasma was approximately 9-fold lower compared with that of tacrolimus (0.4% ± 0.1% vs. 3.7% ± 0.8%).

### 2.5. Sucralose

**Figure 8:**
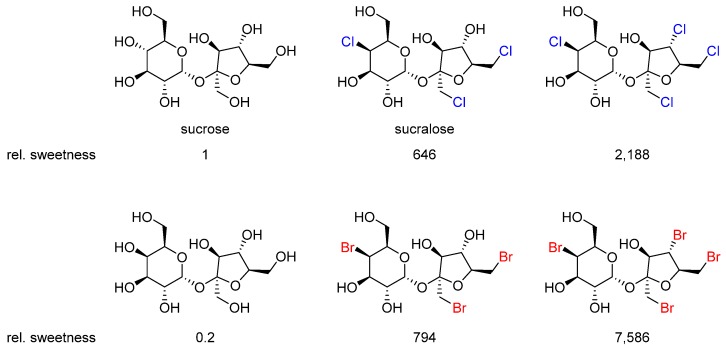
The relative sweetness of some disaccharides and halogenated derivatives. Increased hydrophobicity increases perceived sweetness.

Sucralose (Splenda^®^) is one of the most common artificial sweeteners and sugar substitutes (Figure 8) [29]. The majority of ingested sucralose is not metabolized by the body, which makes it safe and also noncaloric [30]. This example in particular emphasizes the metabolic stability of alkyl chlorides, especially when they are adjacent to other electron-withdrawing heteroatoms (even primary halides can be stable).

Researchers have been investigating the structural requirements for sweetness since the 1960s. While taste sensing is a complex phenomenon, some general rules have been established for which most sweet-tasting compounds adhere to. Schallenberger and Acree proposed that for a compound to be sweet, it needs to possess a hydrogen bond donor, and a Lewis basic site roughly 0.3 nm apart [31,32]. For enhanced sweetness compared to sucrose, the importance of a third, hydrophobic, site was established [33]. In sucralose, two chlorine atoms present in the fructose portion of the molecule comprise the hydrophobic site.

Generally, highly intense sweeteners tend to be more hydrophobic, which is suggested to give rise to stronger absorption to taste bud tissue in contrast to simple sugars, which are more hydrophilic, less sweet and weakly absorbed to the taste buds. This trend can be clearly observed in the series of synthetic halogenated analogs of sucrose (Figure 8) [29].

## 3. Alkyl Halide Natural Products

Natural products have always inspired the development of novel medicines, as exhibited by the fact that between 1981 and 2014 approximately 40% of all new approved drugs were based on natural products [34]. It is therefore instructive to describe the biological activity of some halogenated secondary metabolites that may serve as drug candidates. It is an interesting historical note that for a long time the chemistry community believed very few halogenated natural products existed. However, recent years have witnessed a surge in the isolation of halogenated molecules from marine organisms, counting more than 5000 today [35]. This, in time, could thus translate into a greater number of alkyl chloride and bromide containing drug candidates.

### 3.1. Halomon

**Figure 9:**
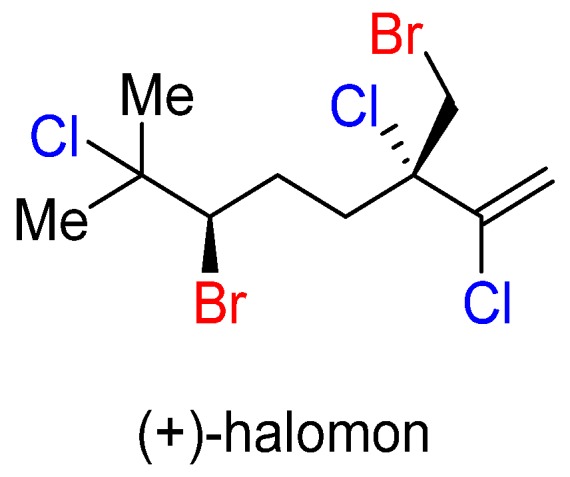
The structure of (+)-halomon, a pentahalogenated myrcene derivative.

Halomon (Figure 9) was isolated in 1975 from the red algae *Portieria hornemannii* [36]. Seventeen years later it produced one of the most extreme cases of differential cytotoxicity in a National Cancer Institute NCI-60 human tumor cell line screen [37]. The compound was selected for preclinical drug development, but then later was dropped due to lack of material [38]. Even though the detailed mechanism of action is unknown, it has been suggested that the activity is due to the inhibition of DNA methyltransferase, an important target for combatting cancer because of its tendency to hypermethylate tumor suppressing regions in tumor suppressing cells [39]. Computational analysis of the cytotoxicity profile of halomon has suggested that the activity cannot be attributable to simple nonspecific alkylation reactions in the body [40]. Investigations into the mechanism of action of this molecule are ongoing within our laboratory through collaborations [41]. Initial results suggest that the unnatural enantiomer, (−)-halomon, is inactive against a series of cancers that are sensitive to (+)-halomon [42].

### 3.2. Lapachone

**Figure 10:**
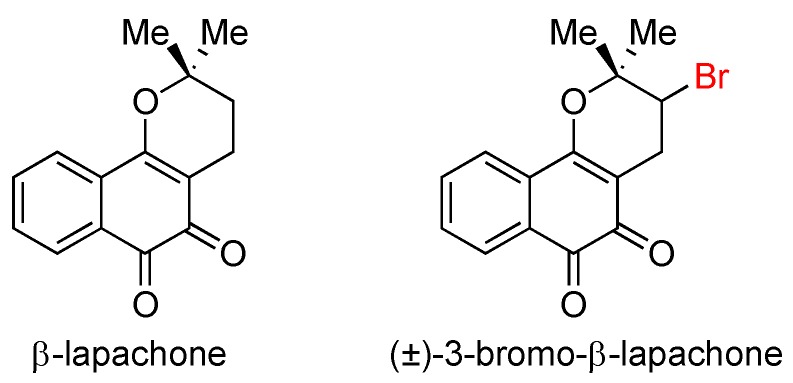
The structures of β-lapachone and its more active analog, 3-bromo-β-lapachone.

β-lapachone is a natural product present in the bark of the lapacho tree, which grows predominantly in Brazil (Figure 10). It is endowed with a large spectrum of pharmacological activities, including antibacterial [43], antifungal [43], and antimalarial [44,45] activities. It is also a potential treatment for prostate and non-small cell lung cancers that has been evaluated in clinical trials [46].

When testing analogs of β-lapachone, researchers discovered that bromination at the C3 position enhances antiplasmoidal activity [45] (*IC*_50_ 2.7 μM vs. 4.1 μM against Plasmodium falciparum, strain F 32) and cytotoxicity [47] (*IC*_50_ 0.13 μM vs. 0.27 μM against promyelocytic leukemia HL-60 cell lines, MTT assay). To date, no study has reported testing of individual enantiomers, nor chlorinated derivatives.

### 3.3. Astins

**Figure 11:**
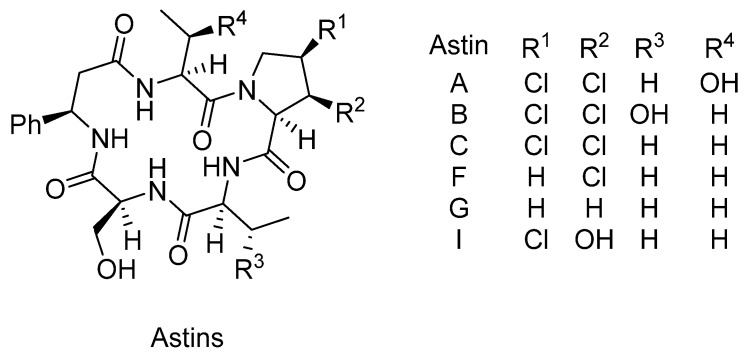
The general structure of compounds belonging to the family of astins.

Astins A–I are a family of cyclic pentapeptides isolated from the medicinal plant *Aster tataricus* (Figure 11). They contain a 16-membered ring of several non-coded amino acids, including a β,γ-dichlorinated proline residue [48]. Their antitumor activity was assayed using Sarcoma 180 ascites in mice. The effectiveness was evaluated in terms of the tumor growth ratio. At 5 mg/kg/day astins A, B and C gave the GR (tumor growth ratio) values of 40%, 26% and 45%, respectively, whereas the other natural astins (F, G, I) and the derivatives of dechlorinated proline residues did not inhibit the tumor growth at 10 mg/kg/day. The presence of *cis* dechlorinated proline residues was concluded to be an important structural motif for astins for antitumor activity.

### 3.4. Forazoline

**Figure 12:**
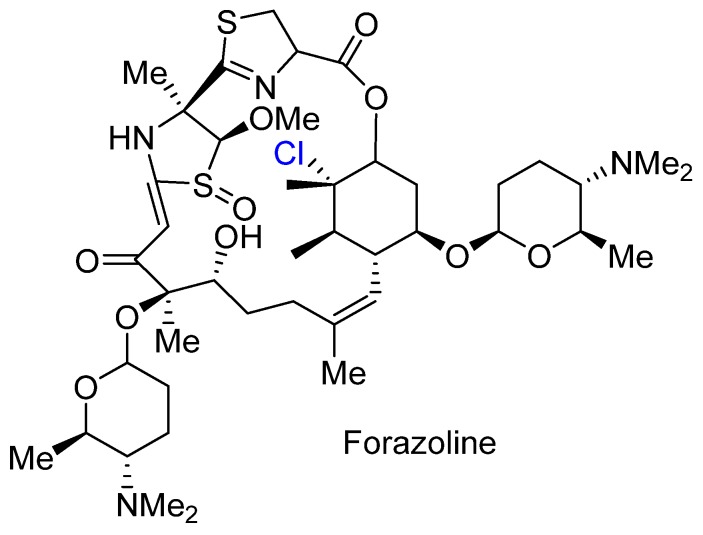
Structure of the recently isolated forazoline A.

Forazoline A was recently isolated from the marine *Actinomadura* sp. (Figure 12) [49]. It demonstrated in vivo antifungal efficacy, comparable to amphotheracin B, against *C. albicans*. It was also found to be nontoxic in mice. Further experiments suggested that forazoline affects cell membranes, potentially through the disregulation of phospholipid homeostasis. It is unknown at this stage how much of the biological activity is attributable to the presence of the alkyl chloride moiety.

## 4. Summary and Outlook

This review has aimed to illustrate the medical benefit of chiral alkyl halides, as well as the features of these compounds that play a key role in their activity. Alkyl halides, especially chlorides, are often stable, which is underscored by the number of compounds in the clinic and food industry containing these motifs. Halogenation, and the stereochemistry of the halogen-bearing carbons can significantly alter bioactivity. This can happen through increased lipophilicity, which leads to better pharmacokinetic properties. However, halogen atoms can also play key roles in binding through halogen bonding interactions, and, in a number of cases, the exact roles of halogens are not fully elucidated. Finally, a growing number of isolated halogenated natural products endowed with unique bioactivity show promise for further development. Looking forward, it is clear that the synthetic community has a huge opportunity for the understanding and prediction of the properties of chiral alkyl halides through molecular design. Looking forward, it is also clear that in order to better understand and predict the properties of chiral alkyl halides, the synthetic community needs novel, highly selective methods for their installation.

## Figures and Tables

**Table 1:** Modification of lincomycin at the C-7 site. All results are MIC (mg/L).

Compound	*Straph. aureus*	*Str. faecalis*
R_1_ = OH, R_2_ = H	0.4	12.5
R_1_ = H, R_2_ = OH	1.6	25
R_1_ = Cl, R_2_ = H	0.8	12.5
R_1_ = H, R_2_ = Cl	0.1	6.2
R_1_ = H, R_2_ = Br	0.05	6.2
R_1_ = H, R_2_ = I	0.05	3.2
